# Danzhi Xiaoyao Powder Promotes Neuronal Regeneration by Downregulating Notch Signaling Pathway in the Treatment of Generalized Anxiety Disorder

**DOI:** 10.3389/fphar.2021.772576

**Published:** 2021-11-29

**Authors:** Chao Liu, Zhenhao Ying, Zifa Li, Long Zhang, Xin Li, Wenbo Gong, Jiang Sun, Xuejing Fan, Ke Yang, Xingchen Wang, Sheng Wei, Ning Dong

**Affiliations:** ^1^ The First Clinical Medical College, Shandong University of Traditional Chinese Medicine, Ji’nan, China; ^2^ School of Rehabilitation Science, Shandong University of Traditional Chinese Medicine, Ji’nan, China; ^3^ Experimental Center, Shandong University of Traditional Chinese Medicine, Ji’nan, China; ^4^ Key Laboratory of Traditional Chinese Medicine Classical Theory, Ministry of Education, Shandong University of Traditional Chinese Medicine, Ji’nan, China; ^5^ Shandong Provincial Key Laboratory of Traditional Chinese Medicine for Basic Research, Shandong University of Traditional Chinese Medicine, Ji’nan, China; ^6^ College of Traditional Chinese Medicine, Shandong University of Traditional Chinese Medicine, Ji’nan, China; ^7^ Department of Neurology, The Second Affiliated Hospital of Shandong University of Traditional Chinese Medicine, Ji’nan, China; ^8^ The Second Clinical Medical College, Shandong University of Traditional Chinese Medicine, Ji’nan, China

**Keywords:** Generalized anxiety disorder, DZXYS, hippocampus, Notch, neuron regeneration

## Abstract

**Background:** Generalized anxiety disorder (GAD) is one of the most common types of anxiety disorders with unclear pathogenesis. Our team’s previous research found that extensive neuronal apoptosis and neuronal regeneration disorders occur in the hippocampus of GAD rats. Danzhi Xiaoyao (DZXYS) Powder can improve the anxiety behavior of rats, but its molecular mechanism is not well understood.

**Objective:** This paper discusses whether the pathogenesis of GAD is related to the abnormal expression of Notch signal pathway, and whether the anti-anxiety effect of DZXYS promotes nerve regeneration in the hippocampus by regulating the Notch signaling pathway.

**Methods:** The animal model of GAD was developed by the chronic restraint stress and uncertain empty bottle stimulation method. After the model was successfully established, the rats in the model preparation group were divided into the buspirone, DZXYS, DZXYS + DAPT, and model groups, and were administered the corresponding drug intervention. The changes in body weight and food intake of rats were continuously monitored throughout the process. The changes in anxiety behavior of rats were measured by open field experiment and elevated plus-maze test, and morphological changes and regeneration of neurons in the rat hippocampus were observed by HE staining and double immunofluorescence staining. Changes in the expression of key targets of the Notch signaling pathway in the hippocampus were monitored by real-time fluorescence quantitative PCR and western blotting.

**Results:** In this study, we verified that the GAD model was stable and reliable, and found that the key targets of the Notch signaling pathway (Notch1, Hes1, Hes5, etc.) in the hippocampus of GAD rats were significantly upregulated, leading to the increased proliferation of neural stem cells in the hippocampus and increased differentiation into astrocytes, resulting in neuronal regeneration. DZXYS intervention in GAD rats can improve appetite, promote weight growth, and significantly reverse the anxiety behavior of GAD rats, which can inhibit the upregulation of key targets of the Notch signaling pathway, promote the differentiation of neural stem cells in the hippocampus into neurons, and inhibit their differentiation into astrocytes, thus alleviating anxiety behavior.

**Conclusion:** The occurrence of GAD is closely related to the upregulation of the Notch signaling pathway, which hinders the regeneration of normal neurons in the hippocampus, while DZXYS can downregulate the Notch signaling pathway and promote neuronal regeneration in the hippocampus, thereby relieving anxiety behavior.

## 1 Introduction

Generalized anxiety disorder (GAD) is a chronic anxiety disorder characterized by persistent significant tension, accompanied by autonomic nervous function excitement and over alertness. According to the World Health Organization (WHO) World Mental Health Survey, anxiety disorder is the most common cause of mental illness and disability worldwide, and generalized anxiety disorder (GAD) is one of the most common types of anxiety disorders (Demyttenaere et al., 2004). The global prevalence of anxiety disorder was 7.3% (Stein et al., 2017). The lifetime prevalence of GAD ranged from 0.8 to 5.1% (Kessler, 2000; Hidalgo and Sheehan, 2012), seriously affecting the quality of life of patients. At the same time, GAD is a risk factor for many other diseases, such as alcohol dependence and drug dependence (benzodiazepine dependence) (Tyrer and Baldwin, 2006). Although GAD is common in clinics, it has not been studied as much detail in neurobiology as many other emotional disorders such as depression (Menard et al., 2016), and its pathogenesis remains to be unclear. At present, it is generally believed that its pathogenesis is closely related to neurotransmitter disorders, and neurotransmitter regulating drugs are often used as treatment, and their effects are not ideal and side effects may be obvious, such as drowsiness, dizziness, nausea, and addiction, etc. (Bandelow, 2020).

Traditional Chinese medicine has been widely used in the treatment of emotional diseases for thousands of years. Danzhi Xiaoyao Powder is a classic Chinese prescription that has been used in the clinic. It originated from Xue Ji’s Internal Medicine Abstract during the Ming Dynasty. It was modified by the “Xiaoyao Powder.” The entire prescription consisted of 10 g danpi, 10 g gardenia, 15 g bupleurum, 10 g Poria cocos, 15 g fried Atractylodes macrocephala, 10 g Angelica sinensis, 10 g white peony, 10 g licorice, 3 g mint, and 9 g ginger, which is widely used in clinical and experimental research on anxiety and depression disorder (Xu et al., 2020). Our team’s previous research found extensive neuronal apoptosis and neuronal regeneration disorders in the hippocampus of GAD rats. DZXYS can improve the anxiety behavior of GAD rats, but its mechanism remains to be explored (Dong, 2015).

In recent years, some scholars have found that the occurrence of depression is related to the decline of adult hippocampal neurons, and stimulating hippocampal neurons is a new strategy for antidepressant treatment (Tanti and Belzung, 2013; Hill et al., 2015; Anacker et al., 2018). The hippocampus has become the focus of research on mental diseases because of its nerve regeneration function and unique regulation of emotion and cognition (Toda et al., 2019). In view of the similarities between depression and anxiety disorders in pathophysiological mechanisms and neuroanatomical changes (Hettema, 2008), we speculate that neuronal apoptosis and neuronal regeneration disorder in the hippocampus of GAD rats found in the previous team’s research are also important pathological mechanisms leading to GAD. Hippocampal newborn neurons do not appear spontaneously; they are the result of proliferation and differentiation of neural stem cells in the granular cell layer (subgranular cells of the dentate gyrus, SGZ) of the hippocampal dentate gyrus (Aguilar-Arredondo et al., 2015; Zupanc et al., 2019). The maintenance of neural stem cell proliferation and its differentiation direction and timing are regulated by the Notch signaling pathway (Lutolf et al., 2002; Niessen and Karsan, 2007), which are expressed in the two neurogenic sites of the SGZ and the subventricular zone (SVZ) in the adult brain (Stump et al., 2002), and participate in adult neurogenesis. The activated Notch signaling pathway can maintain the cellular characteristics of NSCs, inhibit their fate of neuronal differentiation, and promote their differentiation into astrocytes (Guo et al., 2009; Snyder et al., 2012). Astrocytes are the type with the largest number of glial cells, and the ratio of their number to the number of neurons is 3:1 in rodents and 1.4:1 in the human central nervous system (Sofroniew and Vinters, 2010). Under normal conditions, astrocytes can regulate the concentration of ions inside and outside neurons, nutritional repair, synaptic transmission, and construct a neural tissue grid, forming the blood-brain barrier; however, excessive proliferation can form glial scars, secrete nerve axon regeneration inhibitors, inhibit neuron and nerve axon regeneration, and lead to nerve regeneration disorder (Pekny et al., 2007; Singh and Joshi, 2017). We speculate that the Notch signaling pathway plays an important role in the pathogenesis and treatment of GAD. Therefore, this manuscript discusses the role of Notch signaling pathway in the pathogenesis of GAD and whether the anti-anxiety effect of DZXYS is related to the regulation of Notch signaling pathway to promote nerve regeneration in the hippocampus and improve anxiety behavior (Ren and Zuo, 2012).

## 2 Materials and Methods

### 2.1 Animal Preparation

All the experiments were approved by the ethics review committee of Shandong University of Traditional Chinese Medicine (No. DWSY201703013) and carried out in accordance with “the Guidelines for the Care and Use of Experimental Animals of the National Institutes of Health”. All the studies attempted to reduce the pain felt by the animals. Animals: Male Wistar rats (*n* = 120, 6 weeks old, 200 ± 10 g) were purchased from Beijing Vital River Experimental Animal Technology Co., Ltd (Beijing, people’s Republic of China; No. SCXK 2016–0006). During the standard 12 h light/dark cycle, the animals were kept at a room temperature of 21 ± 1°C and relative humidity of 30–40%. After 1 week of adaptation, the rats were randomly divided into two groups according to their body weight: control group (*n* = 24) and model preparation group (*n* = 96). The control group did not have a model, a free diet, or drinking water.

### 2.2 GAD Modeling Method

**FIGURE 1:**
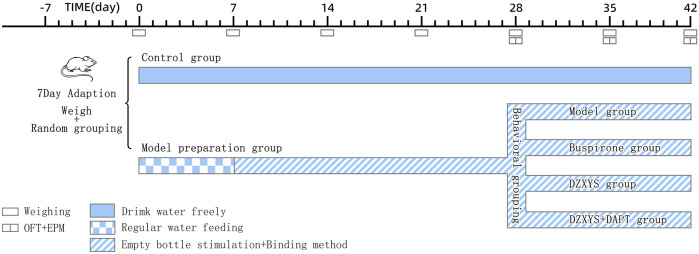
Schedule for the model preparation protocol and behavioural testing. OFT, open field test; EPM, elevated plus-maze test.

The GAD model was established in the model preparation group according to the uncertain empty bottle stimulation method and chronic restraint stress. The specific methods were as follows: first, 7 days of regular water feeding training, the rats were given a water bottle filled with water at 9:00–9:10 and 21:00–21:10 every day to drink for 10 min, and the rest of the time spent on removing the water bottle without water, so that the rats were in a thirsty state. After the rats were used to drinking water at two time points every day, from the 8th day to the 28th day, randomly select one time period to feed water to the water bottle for 10 min, and the other time period to stimulate the rats with empty water bottle, observe the rats’ behavior of attacking the cage, looking around the water bottle and modification behavior, etc. At the same time, from 9:30 am to 15:30 pm every day, the rats were fixed with tubular rat fixator (Model: HL-DTS, Specification: 20*6 cm, Material: plexiglass, Heli Kechuang Technology Development Co., Ltd, Beijing) for 6 h for 21 days. On the 28th day, the elevated plus maze (EPM) and open field test (OFT) behavioral tests were performed after restraint, and the rats in the model preparation group were divided into DZXYS group, DZXYS + DAPT group, buspirone group, and model group with the time for rats to open arms in the EPM as the baseline, with 24 rats in each group. Different drug interventions were performed. During the drug intervention, the uncertain empty bottle stimulation and restraint methods were still carried out synchronously until the end of the drug intervention. From the beginning of the experiment to the 42nd day, the changes in body weight and food intake were measured every week (Figure 1).

### 2.3 Drug Preparation

The traditional Chinese medicine compound used in the experiment was Danzhi Xiaoyao Powder Formula Granule (1,035,073, Yifang Pharmaceutical Co., Ltd., Guangzhou, China). The granule was dissolved in the distilled water at a concentration of 1.05 g/ml. The drug concentration has been verified by a previous experiment conducted by the research group, and the curative effect is ideal (Dong Ning, 2015). The dose of Buspirone hydrochloride tablets (H19991024, Enhua Pharmaceutical Co., Ltd., Jiangsu, Beijing) were calculated to be 7 times the adult dosage (20 mg/60 kg/d), i.e., the dosage of rats was 2 mg/kg/d, which was dissolved in distilled water at a concentration of 0.2 mg/ml (Garakani et al., 2020). The drug has no addiction and has good anti-anxiety effects and has been found to promote neuronal regeneration in the hippocampus as a 5-HT1A receptor agonist (Masayoshi et al., 2014). 5-Bromo-2-deoxyuridine (BrdU Sigma Chemical Company, St. Louis, MO) was injected intraperitoneally into rats to label proliferating cells. BrdU solution was prepared from 0.9% NS at a concentration of 15 mg/ml and a rat injection dose of 50 mg/kg/time.DAPT (r-secret inhibitor) (D7230, Solarbio, Beijing, China)is a Notch signaling pathway inhibitor, dissolve 50 mg DAPT in 1 ml DMSO before use, and then add 0.9% NS to dilute it into a solution with a concentration of 4.54 mg/ml, and the dosage of intraperitoneal injection was 20 mg/kg/d (Breunig et al., 2007; Zhang et al., 2019).

In this experiment, only Notch signaling pathway inhibitors were used without agonists, for two reasons. Firstly, by consulting the literature, we have learned that the activated Notch signaling pathway can maintain the cellular characteristics of NSCs and inhibit their fate of differentiation into neurons, so as to promote the differentiation into astrocytes (Guo et al., 2009; Snyder et al., 2012). We speculate that excessive upregulation of the Notch signaling pathway is an important reason for the excessive proliferation and differentiation of neural stem cells into astrocytes, resulting in the obstacle of neuronal regeneration, and the application of Notch signaling pathway inhibitors can often inhibit the excessive proliferation of neural stem cells and restore the normal differentiation of neural stem cells into neurons (Breunig et al., 2007). If the application of the signal pathway inhibitor can help Chinese medicine reverse anxiety behavior, it confirms our hypothesis, so as to avoid excessive sacrifice of experimental animals, which is also in line with our animal ethical practices. In addition, the commonly used and stable Notch signaling pathway agonists are Notch signal pathway ligands such as Jagged1 and Dll4 (Saffarzadeh et al., 2019). Another important topic of our team was to explore whether DZXYS can regulate Notch ligands. If such agonists are used, our research will be disturbed.

### 2.4 Drug Administration

**FIGURE 2:**
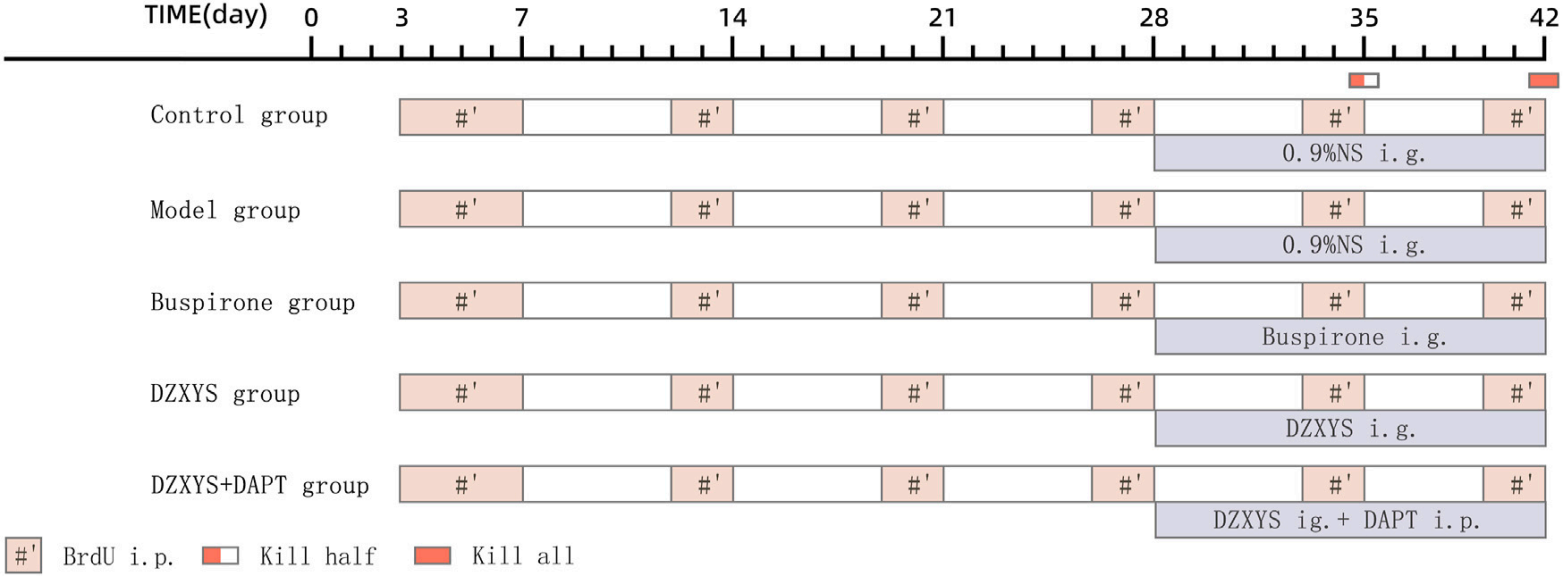
Schedule for the drug administration. Abbreviations: i. p, intraperitoneal injection; ig, intragastric administration.

From the 28th day after successful modeling, the rats in each group were given an oral stainless steel gavage needle 30 min before stimulation with an uncertain empty bottle of water every day. The DZXYS, DZXYS + DAPT and buspirone groups were given DZXYS solution and buspirone solution respectively according to the dosage of 1 ml/100 g body weight, and the blank group and model group were also given 0.9% NS according to 1 ml/100 g body weight, lasting for 14 days. In addition, rats in the DZXYS + DAPT group were intraperitoneally injected with DAPT and the rats in each group were injected intraperitoneally with BrdU during the modeling period. The injection frequency was as follows: BrdU was injected intraperitoneally twice a day, 5 days before the start of modeling, and then twice a week. One day before death, the rats were injected every 8 h three times (Figure 2).

### 2.5 Animal Behavior Test

Open-field test: The OFT experiment was conducted on the 28th day after modeling, 7 days after intragastric administration (35th day), and 14 days after administration (42nd day). The animals were placed in a quiet test room illuminated by red light for 30 min in advance to adapt to the environment. The open field is a black square device (length*width*height is 100 cm*100 cm*50 cm), and the bottom of the field is divided into nine equal squares. Each rat was placed separately in the center of the site and allowed to explore freely for 6 min. A camera was installed directly above the open field and connected to a computer on the side. The XR super-maze tracking system records the total distance, speed, time, number of times entering the central area, number of times standing up, and the number of times the horizontal grids were crossed (Ennaceur et al., 2009). After each rat was tested, the site was disinfected with 75% ethanol to remove any residual odor. When the animals stayed around the open field for a significantly longer time than in the central area, it proved that the rats had anxiety tendencies.

Elevated Plus Maze: The experiment was conducted in a quiet room illuminated by red light. EPM is a polypropylene plastic cross-shaped device 76 cm above the ground and consists of two closing arms (50 × 10 cm), two open arms (50 × 10 cm), and a central platform (10 × 10 cm). We put the rat’s head towards the closed arm on the central platform, and used the XR-super Maze tracking system to record the action track of rats within 6 min, including the frequency (OE) and time (OT) of entering the open arm, and the frequency (CE) and time (CT) of entering the closing arm (Biedermann et al., 2017). OE% and OT% were calculated as follows: OE% = OE/(OE + CE) × 100% and OT% = OT/(OT + CT) × 100%. If the OE% and OT% of rats were low, the anxiety behavior of rats was obvious.

### 2.6 Sample Collection and Preparation

Pentobarbital (2%) was injected into the abdominal cavity of rats to induce deep anesthesia (40 mg/kg). Some rats were decapitated, and their brains were removed. The hippocampus in the brain tissue was quickly stripped and stored in liquid nitrogen for subsequent western blotting and RT qPCR detection. The remaining rats in each group were perfused with 0.9% NS 500 ml and 4% paraformaldehyde 150 ml, the brain tissue was quickly removed and soaked in 4% paraformaldehyde, fixed for 24 h, and the area of the hippocampus was trimmed into small pieces with a thickness of 5 mm. The small pieces were dehydrated with gradient concentration of ethanol (75, 85, 95% for 2 h, 100% for 1 h), transparent in xylene (45 min), and soaked in paraffin (three times, 1 h each time). The tissues were embedded in an embedding machine to make paraffin blocks (Mohamed et al., 2017). Each paraffin block was cut into four 4 um pathological sections using a Leica paraffin slicer (Histocore Biocut Paraffin Slicer, Leica, United States).

### 2.7 HE Staining

First, paraffin sections of brain tissue from each rat were taken, dewaxed and hydrated, washed with 0.1 M PBS three times, then immersed in hematoxylin dye for 5 min, washed with tap water after removal, differentiated with 1% hydrochloric acid alcohol for several seconds, returned to blue with 0.6% ammonia, and rinsed with running water for 5 s. They were dyed in eosin solution for 2 min and rinsed with running water. Finally, the slices were successively placed in gradient alcohol and xylene, dehydrated, transparent, and sealed with neutral gum (Feldman and Delia, 2014).

### 2.8 Double Immunofluorescence

Three paraffin sections were obtained from the brain tissue of each rat. Each section was double-stained with anti-BrdU/anti Nestin (anti-BrdU mouse mAb, GB12051, Servicebio; Anti-Nestin Mouse mAb, GB12137, Servicebio), anti-BrdU/anti NeuN (Anti-NeuN Mouse mAb, GB11138, Servicebio), and anti-BrdU/anti GFAP (anti-GFAP rabbit pAb, GB11096, Servicebio). The staining steps of immunofluorescence double staining are briefly described as follows: First, the sections were dewaxed and hydrated, repaired with EDTA antigen repair solution, and the sections were washed with 0.1 M PBS, and then incubated in goat serum for 30 min. Each slice was then incubated with different pairs of primary antibodies at 4°C overnight. The next day, after recovery to room temperature, they were washed with 0.1 M PBS three times, and mixed secondary antibody (Alexa Fluor^®^ 488 labeled goat anti-rabbit IgG, GB25303, Servicebio; Cy3 labeled goat anti-rabbit IgG, GB21303, Servicebio), incubated in the dark at room temperature for 20 min, washed with 0.1 M PBS for 3 times, Cy3-TSA was added dropwise and incubated at room temperature for 10 min, and the nuclei treated with 4′,6-diamidino-2-phenylindole (DAPI; ThermoFisher Scientific, Waltham, MA, United States). Finally, two drops of fluorescence quencher were added to each slice to seal it and was observed under an upright fluorescence microscope (Leica SP8, Hamburg, Germany)(Mohan et al., 2008; Lightbody and Nicol, 2019).

### 2.9 Real Time-qPCR

Total RNA was extracted from 60 rat hippocampal tissues using Trizol (CWBIO, CW0580s) according to the manufacturer’s instructions. A large amount of cDNA was obtained using a HiFiScript cDNA Synthesis Kit. In the ultrasound mixture real-time fluorescence quantitative PCR system, the detection of fluorescence quantitative PCR is efficient and sensitive. Using 2^−ΔΔCT^, the relative expression of each gene was calculated by the CT method, and the expression of each protein was standardized by the GAPDH housekeeping gene (Derveaux et al., 2010).

Gene-specific primers for quantitative RT-PCR included: GAPDH forward, 5-CCT​TCC​GTG​TTC​CTA​CCC​C-3, GAPDH reverse, 5-GCC​CAG​GAT​GCC​CTT​TAG​TG-3; Notch1 forward,5′-TGGATGCCGCTGACCTACG-3, Notch1 reverse, 5-TGG​ATG​CCG​CTG​ACC​TAC​G-3; Hes1 forward, 5′-TTG​AGC​CAA​CTG​AAA​ACA​CTG​ATT- 3′, Hes1 reverse, 5-GTG​CTT​CAC​TGT​CAT​TTC​CAG​AAT-3; Hes5 forward, 5-GAT​GCT​CAG​TCC​CAA​GGA​GAA​AA-3, Hes5 reverse, 5-CCA​CGA​GTA​ACC​CTC​GCT​GTA​GT-3’.

### 2.10 Western Blotting

Hippocampal samples were dissected on ice, homogenized in ice-cold lysis buffer, and the protein concentration of the supernatant was measured on a spectrophotometer. A loading buffer was added to the samples to boil them. Then, 50 ug protein was loaded into 10% Bis-Tris gel, and the strip was transferred to a polyvinylidene fluoride membrane (PVDF) and sealed with sealing solution. The primary antibodies Hes1(1:300) (#11988, CST, American), Hes5 (1:300) (22666-1-AP, Proteintech, China), and Notch1 (1:1000) (#36081, CST, United States) were incubated at 4°C overnight. The secondary antibodies conjugated with horseradish peroxidase were added dropwise on the next day and visualized by enhanced chemiluminescence. The expression of each target gene was standardized using the GAPDH housekeeping gene (Pillai-Kastoori et al., 2020).

### 2.11 Statistical Analysis

The data were analyzed using Graph Pad Prism 7.00 software (Graph Pad Software, Inc, San Diego, California, United States). Outliers were eliminated by the statistical elution method (values that deviate from the mean ±2 times the standard deviation are excluded) (Nakagawa and Cuthill, 2009). The data were expressed as Mean ± SEM. In all group tests, whether using parametric analysis of variance or repeated measurement of parameters, the data before analysis of variance were subject to a normality test (Kolmogorov-Smirnov test) and variance homogeneity test (Levene test). If not, a nonparametric test was used. All data in this study were normal and homogeneous in terms of variance. The transcriptional expression levels of Notch1, Hes1, and Hes5 mRNA and the protein expression levels of Notch1, Hes1, and Hes5 in the hippocampus were analyzed by one-way ANOVA. Repeated measurement two-way ANOVA was used to analyze the food intake, body mass growth rate, total distance of exercise, distance to the central area, residence time in the central area, number of upright, and BrdU+/Nestin+, BrdU+/NeuN+, BrdU+/GFAP + expression in the DG area of the hippocampus. For all analyses, if appropriate, post hoc comparisons were performed using the Bonferroni posthoc tests test. The significance level of ANOVA and post-test was set at *p* < 0.05.

## 3 Results

### 3.1 DZXYS Increased Food Intake and Weight Gain in GAD Rats

**FIGURE 3:**
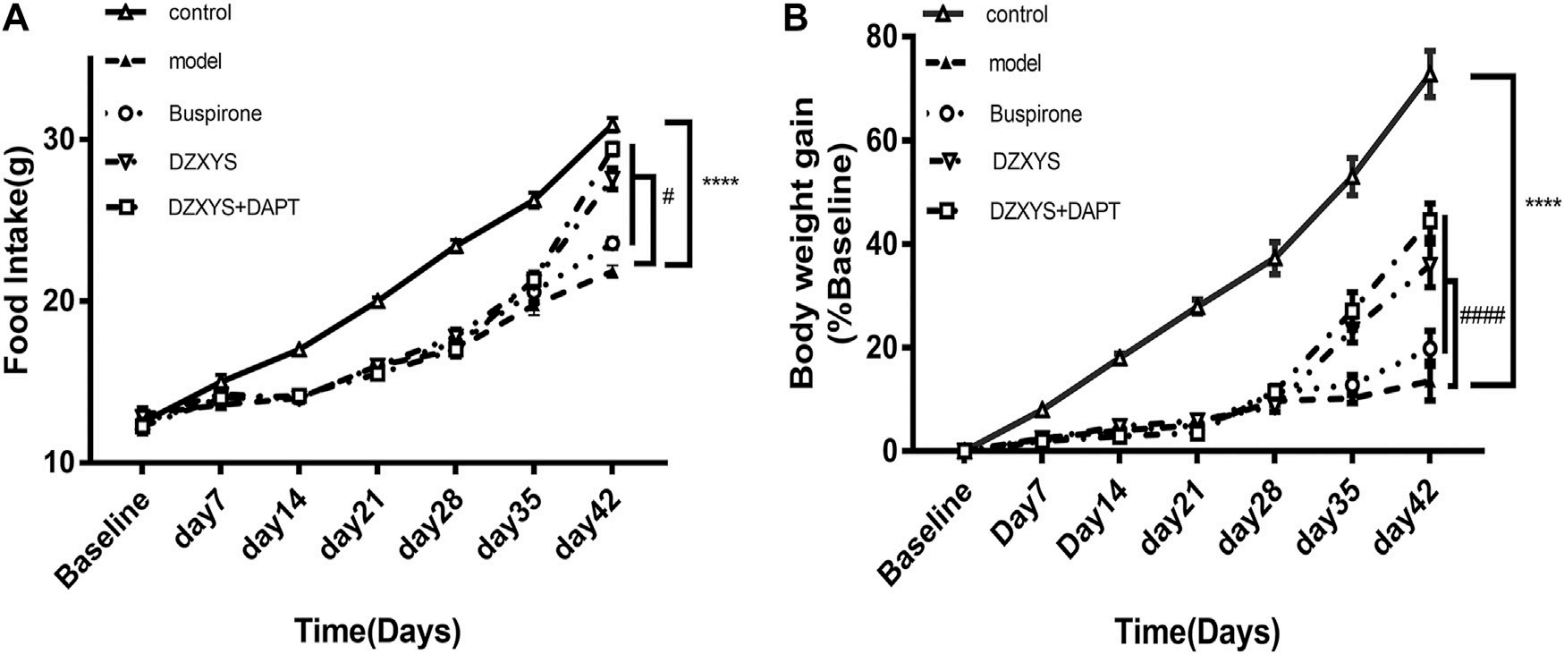
Food intake of rats **(A)** and body weight growth rate **(B)** of rats in each group at the end of stress and drug intervention up to the 42nd day. Control: control group (Hollow triangle); model: model group (Solid triangle); Buspirone: Buspirone group (Hollow circle); DZXYS: DZXYS group (Inverted hollow triangle); DZXYS + DAPT: DZXYS + DAPT Group (Hollow square); ****p < 0.0001 vs control group. #*p* < 0.05, ####*p* < 0.0001 vs model group.

There are significant differences in food intake between groups (Figure 3A).

The Bonferroni post hoc test showed that the model group was significantly lower than the control group (*p* < 0.0001). There was no significant difference between the drug intervention groups and the model group after 7 days of drug intervention. After 14 days of DZXYS and DZXYS + DAPT intervention, the loss of appetite was reversed (*p* < 0.0001 and *p* < 0.0001, respectively). This shows that chronic stress stimulation affects the appetite of rats. The intervention of DZXYS or DZXYS + DAPT for a long time can improve the appetite of rats in the model group. Two-way ANOVA showed a drug treatment effect (F_4,52_ = 90.72, *p* < 0.0001) and time effect (F_6,312_ = 673.5, *p* < 0.0001). The growth rate of the body mass of the rats in each group was significantly different (Figure 3B). On the 42nd day, after stress and drug intervention, there was a significant difference in body mass growth rate between the groups. The Bonferroni post hoc test showed that the model group was significantly lower than the control group (*p* < 0.0001). After intervention with buspirone, DZXYS and DZXYS + DAPT, weight gain retardation was reversed on Day 35 and Day 42 respectively (*p* = 0.0103, *p* < 0.0001, *p* < 0.0001; *p* < 0.0001, *p* < 0.0001, *p* < 0.0001). The results showed that stress had an impact on the normal physiological metabolism of rats, and the three drug interventions corrected the abnormal physiological metabolism of stressed rats to varying degrees. Two-way ANOVA showed significant drug treatment effect (F_4,52_ = 1631, *p* < 0.0001)And time effect (F_6,312_ = 2,700, *p* < 0.0001).

### 3.2 DZXYS Reverses Anxiety Behavior in GAD Rats

**FIGURE 4:**
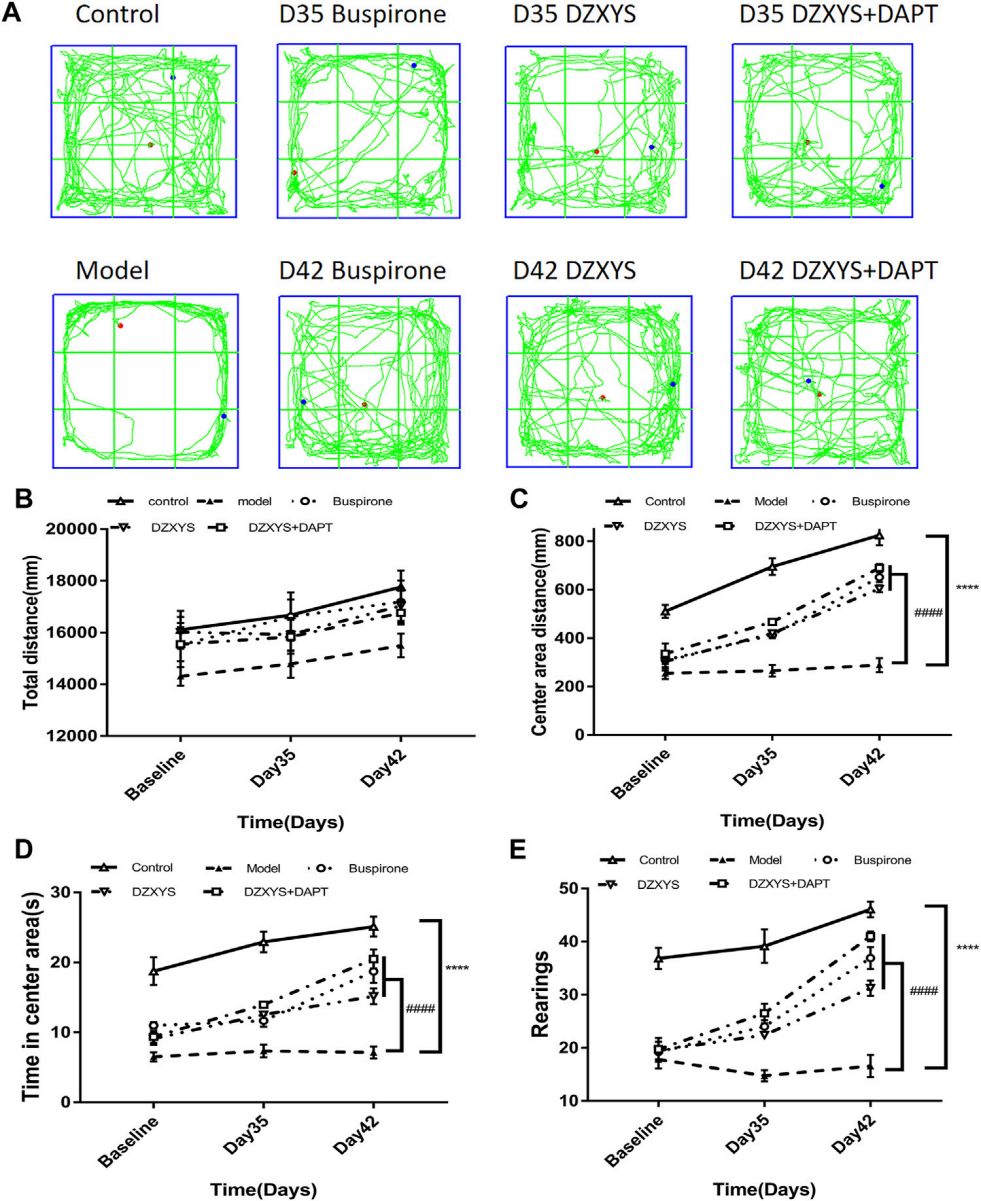
Shows the results of the OFT experiment **(A)** The trajectory of OFT in each group **(B)** Total distance (mm) traveled in a 6 min period **(C)** Center area distance (mm) **(D)** Time in center area(s) **(E)** Number of rearings. ****p < 0.0001 vs control group. ####*p* < 0.0001 vs. model group, repeated measurements of two-way ANOVA.

In order to observe the effect of DZXYS on the improvement of anxiety behavior in GAD rats, we used the internationally widely used OFT and EPM behavioral testing methods to detect the changes in anxiety behavior in rats. As shown in Figure 4B, there was no significant difference in the total movement distance of rats in each group in the open field at each time node. Two-way ANOVA showed no significant treatment effect (F_4,52_ = 1.935, *p* = 0.1184).

There were significant differences among the groups in the central area exercise distance in the open field experiment (Figure 4C). The Bonferroni post hoc test showed that the model group was significantly lower than the control group (*p* < 0.0001), and buspirone, DZXYS, and DZXYS + DAPT were significantly higher than the model group after 7 days (Day 35) and 14 days (Day 42) (*p* = 0.0010, *p* = 0.0009, *p* < 0.0001; *p* < 0.0001, *p* < 0.0001, *p* < 0.0001, respectively). Two-way ANOVA showed that the movement distance of the central area had a significant processing effect (F_4,52_ = 63.28, *p* < 0.0001) and time effect (F_2,104_ = 147.2, *p* < 0.0001).

The residence time in the central area of the rats in each group was significantly different between the groups (Figure 4D). The Bonferroni post hoc test showed that the model group was significantly lower than the control group (*p* < 0.0001), and buspirone, DZXYS, and DZXYS + DAPT were significantly higher than the model group on the 35th and 42nd days after intervention (*p* = 0.0377, *p* = 0.0115, *p* = 0.0006; *p* < 0.0001, *p* < 0.0001, *p* < 0.0001, respectively). The treatment effect of residence time in the central area was as significant as the time effect (F_4,52_ = 51.7, *p* < 0.0001) (F_2,104_ = 51.29, *p* < 0.0001). The results showed that the model group rats had obvious anxiety-like behaviors. Buspirone, DZXYS, DZXYS, and DAPT alleviated anxiety-like behavior in stressed rats.

The number of upright times in the open field of rats in each group was significantly different between the groups (Figure 4E). Bonferroni post hoc analysis showed that the model group was significantly lower than the control group (*p* < 0.0001), and buspirone, DZXYS, DZXYS + DAPT were significantly higher than the model group after 7 days (Day 35) and 14 days (Day 42) (*p* = 0.0021, *p* = 0.0252, *p* < 0.0001; *p* < 0.0001, *p* < 0.0001, *p* < 0.0001, respectively). Two-way ANOVA showed that the number of upright rats had a significant treatment effect (F_4,52_ = 60.87, *p* < 0.0001) and time effect (F_2,104_ = 65.22, *p* < 0.0001).

**FIGURE 5:**
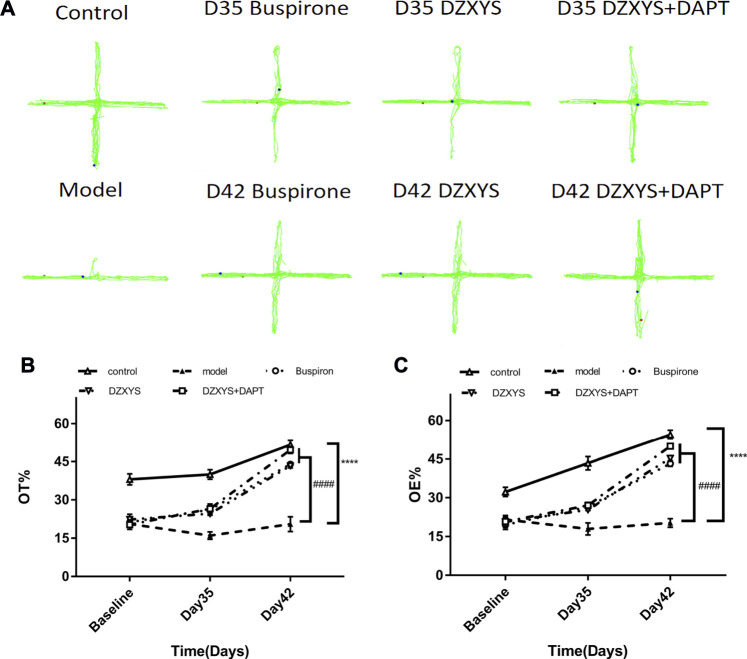
The results of the EPM experiment. **(A)** The movement track of rats in the EPM experimental device. **(B)** The percentage of time in the open arm in the EPM test **(C)** Percentage of open arm entries in the EPM test. ****p < 0.0001 vs control group. ####*p* < 0.0001 vs. model group, two-way ANOVA of repeated data measurements.

The behavioral trajectories of rats in the different treatment groups in the EPM device are shown in Figure 4. In the EPM test, significant differences are observed in the percentage of time entering the open arm (OT%) (Figure 5B) and the percentage of frequency entering the open arm entrance (OE%) (Figure 5C). Bonferroni post-test results showed that the OT% and OE% values of the model group were significantly lower than those of the control group (*p* < 0.0001, *p* < 0.0001), including buspirone and DZXYS after DZXYS + DAPT intervention for 7 days (Day 35) and 14 days (Day 42), The OT% value was significantly higher than that in the model group (*p <* 0.0001, *p* = 0.0046, *p <* 0.0001; *p* < 0.0001, *p* < 0.0001, *p* < 0.0001), and OE% was significantly higher than that in the model group (*p* = 0.0035,*p* = 0.0176, *p* = 0.0048; *p* < 0.0001, *p* < 0.0001, *p* < 0.0001).

Two-way ANOVA showed that OT% had a significant treatment effect (F_4,52_ = 80.39, *p* < 0.0001) and time effect (F_2,104_ = 140, *p* < 0.0001). The OE% value drug treatment effect was as significant as the time effect (F_4,52_ = 85.93, *p* < 0.0001) (F_2,104_ = 174.6, *p* < 0.0001).

### 3.3 DZXYS Improves the Morphology of Hippocampal DG Neurons in GAD Rats

**FIGURE 6:**
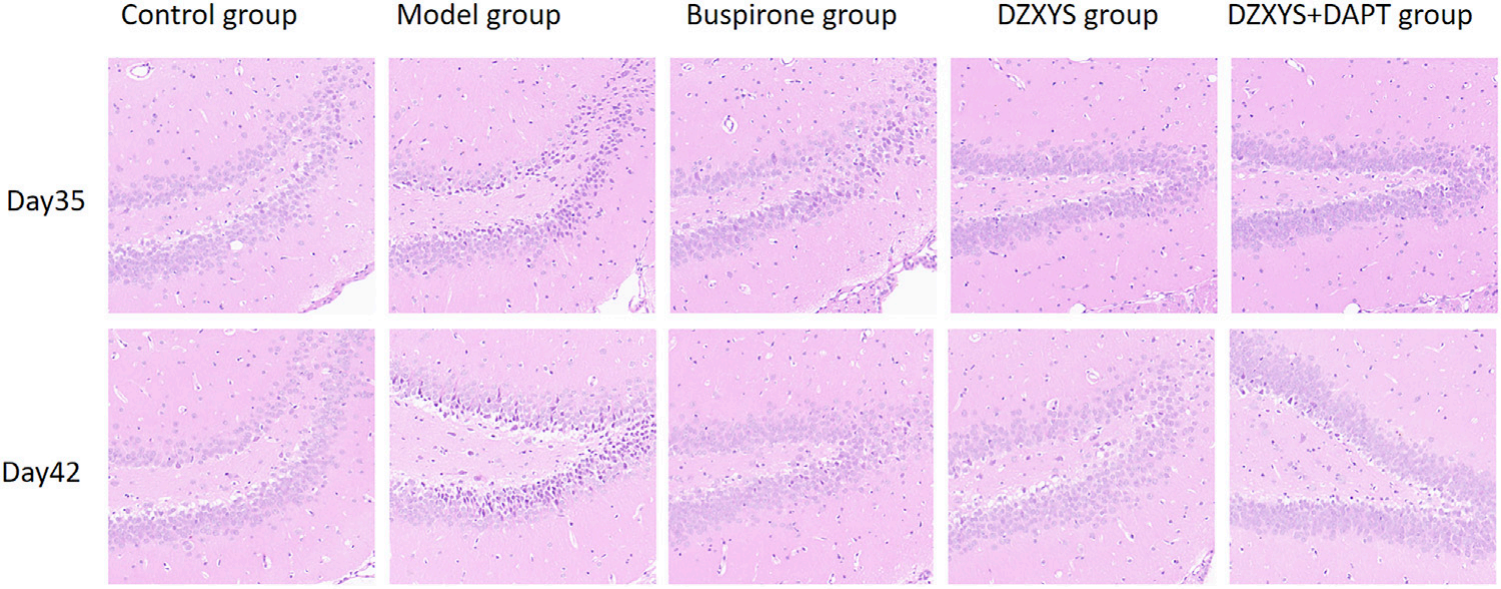
Comparison of HE staining images of neurons in the DG area of the hippocampus in five groups on the 35th and 42nd day after modeling.

We found that after HE staining in the hippocampus of rats in each group (Figure 6), the pyramidal nucleus in the hippocampus of rats in the normal group was large and round, the morphology was intact, the cytoplasm was pink, the nucleus was blue, the chromatin was evenly distributed, the nucleolus was obvious, and the connection between cells was tight and arranged neatly, while in the model group, the number and layers of cells in the hippocampus decreased and were disorderly arranged. It can be seen that a large number of neurons have nuclear chromatin gathering on the lower edge of the nuclear membrane, nuclear pyknosis, deep staining, irregular morphology, cytoplasmic concentration, dark red, and even apoptotic bodies. Neurons lack blindness, the arrangement between cells is disordered, loose, and irregular, and there are gaps around the cells. This is especially true in the DG area of the hippocampus. After 7 days of drug intervention (Day 35), the morphology of neurons in the hippocampus of the buspirone, DZXYS, and DZXYS + DAPT groups was slightly improved compared with that of the model group, but the number of cell layers were still less than that of the control group. Some nuclear chromatin gathered under the nuclear membrane, along with nuclear pyknosis, deep staining, and irregular morphology. After 14 days of drug intervention (Day 42), the morphology of hippocampal neurons in the DZXYS + DAPT and buspirone groups was significantly improved compared to that in the model group. Most cells had a clear outline and were arranged closely. Only a small number of nuclei had slight pyknosis and deep staining of the cytoplasm, which was similar to that in the control group. The morphology of hippocampal neurons in the DZXYS group was further improved compared to that at 7 days, but the arrangement was loose, which was slightly worse than that in the DZXYS + DAPT and buspirone groups.

### 3.4 DZXYS Promotes Neurogenesis in DG Region of Hippocampus in GAD Rats

**FIGURE 7:**
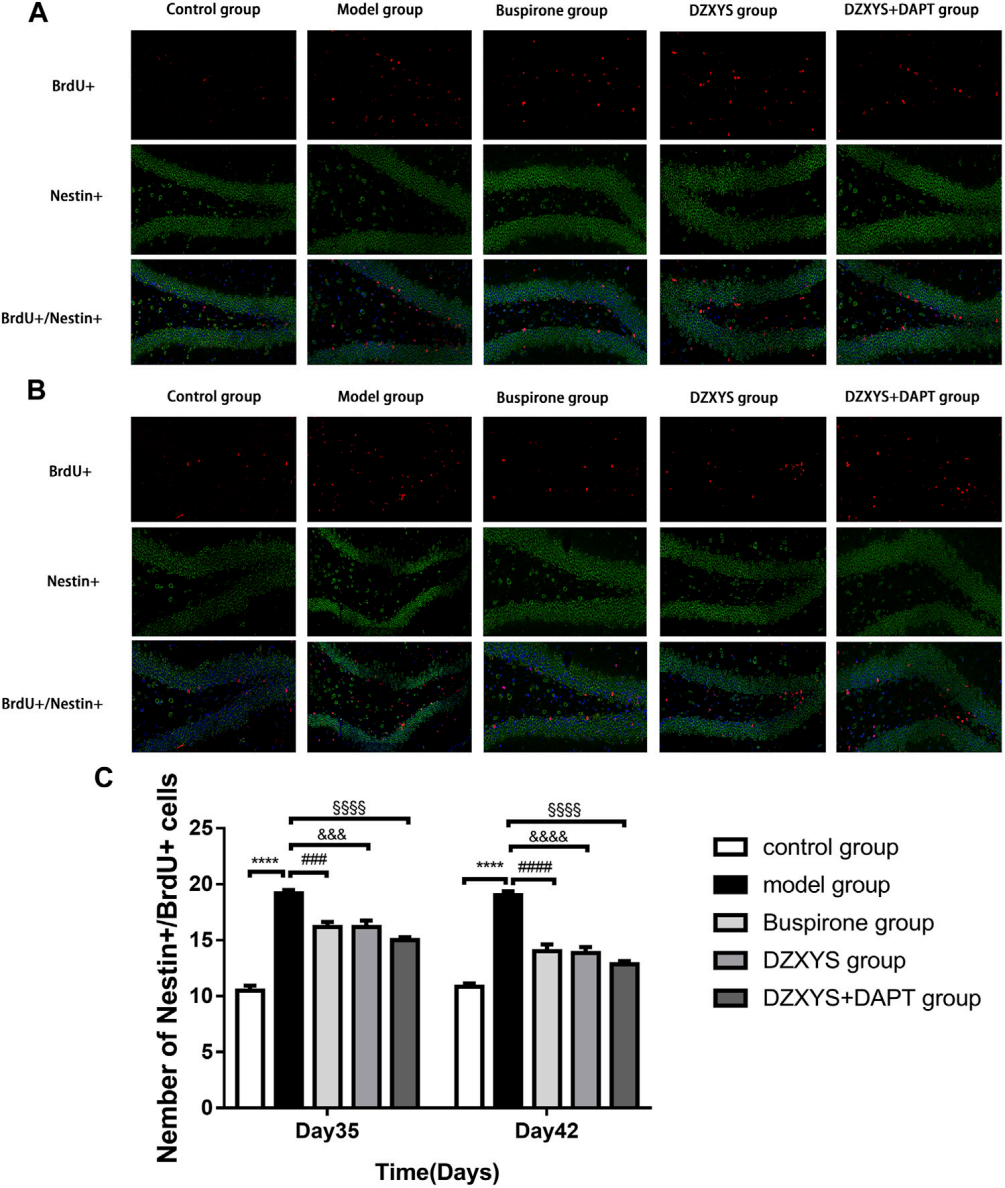
The number of new neural stem cells in the dentate gyrus of hippocampus in each group was shown. BrdU (red) labeled newly formed cells, Nestin (green) labeled neural stem cells. The nuclei were labeled with DAPI (blue) **(A)** On the 35th day, the number of neural stem cells was increased **(B)** On the 42nd day, the number of neural stem cells was increased **(C)** The number of neural stem cell positive cells in dentate gyrus of hippocampus was observed on the 35th and 42nd day.****p < 0.0001, model group vs. control group; ###*p* < 0.001, ####*p* < 0.0001, Buspirone group vs Model group; &&&*p* < 0.001, &&&&*p* < 0.0001, DZXYS group vs. model group; §§§§*p* < 0.0001, DZXYS + DAPT group vs model group.

To observe the proliferation of neural stem cells (NSCs), we labeled newly proliferating cells with BrdU (red) antibody and neural stem cells with nestin antibody (neural progenitor cell-specific marker; green) (Bernal and Arranz, 2018) in the dentate gyrus of rats. There were significant differences in the number of newborn NSCs in the DG area of the hippocampus between the groups (Figures 7A–C). The Bonferroni post hoc test showed that the number of newborn NSCs in the model group was significantly higher than that in the control group (*p* < 0.0001), and the number of buspirone, DZXYS, DZXYS + DAPT on the 35th and 42nd days after intervention was significantly lower than that in the model group (*p* = 0.0004, *p* = 0.0004, *p* < 0.0001; *p* < 0.0001, *p* < 0.0001, *p* < 0.0001, respectively). Two-way ANOVA showed that the number of neonatal NSCs in the DG area of rats had a significant treatment effect (F_4,20_ = 52.16, *p* < 0.0001) and time effect (F_1,20_ = 5.488, *p =* 0.0296).

**FIGURE 8:**
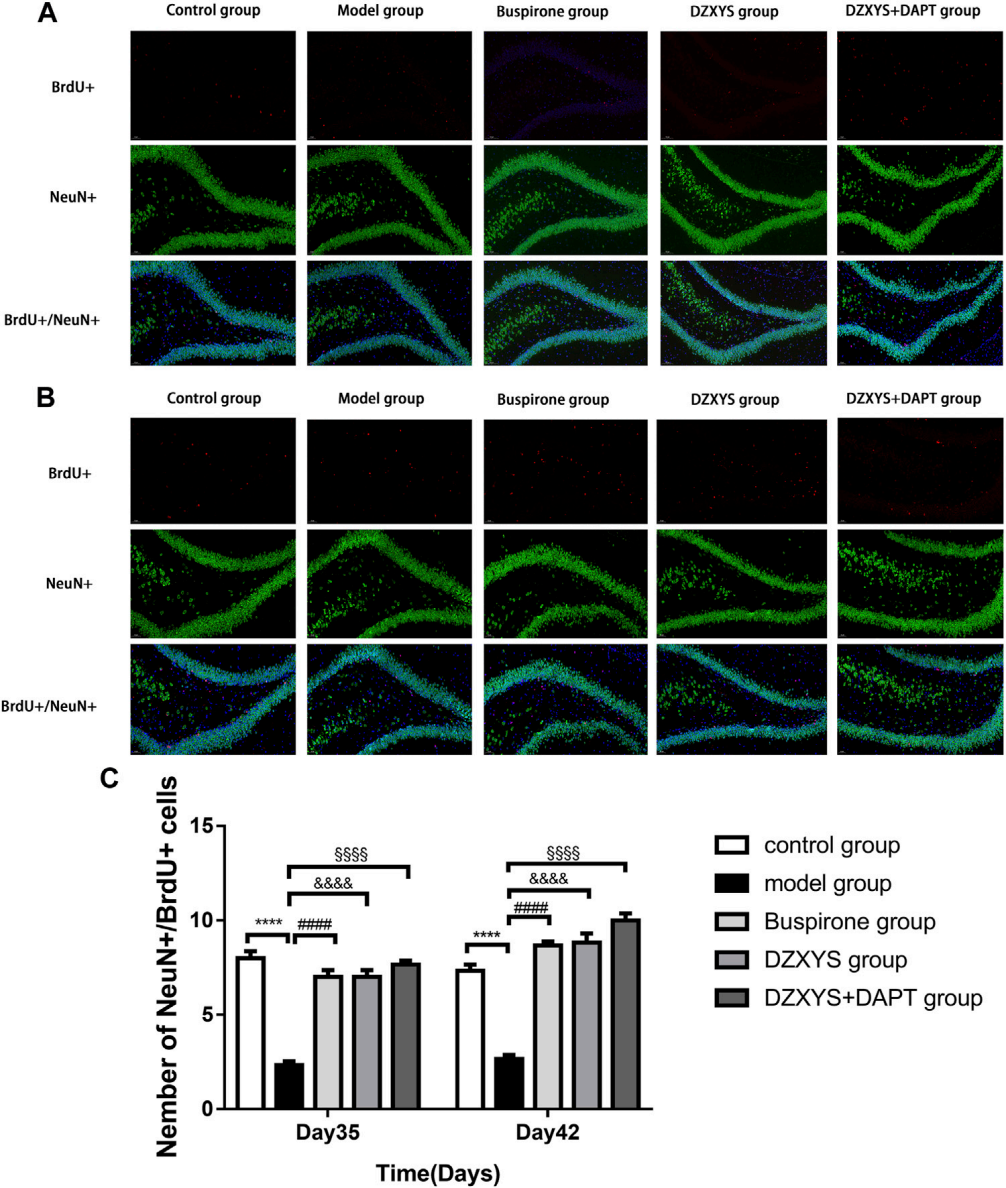
The number of new neurons in the dentate gyrus of the hippocampus in each group. BrdU (red) labeled newly formed cells and NeuN (green)-labeled neurons. The nuclei were labeled with DAPI (blue) **(A)** The regeneration of neurons was observed on the 35th day **(B)** Regeneration of neurons on day 42 **(C)** The number of newborn neurons in the dentate gyrus of the hippocampus on the 35th and 42nd day. ****p < 0.0001, model group vs. control group; ####*p* < 0.0001, Buspirone group vs Model group; &&&&*p* < 0.0001, DZXYS group vs. model group; §§§§*p* < 0.0001, DZXYS + DAPT group vs model group.

**FIGURE 9:**
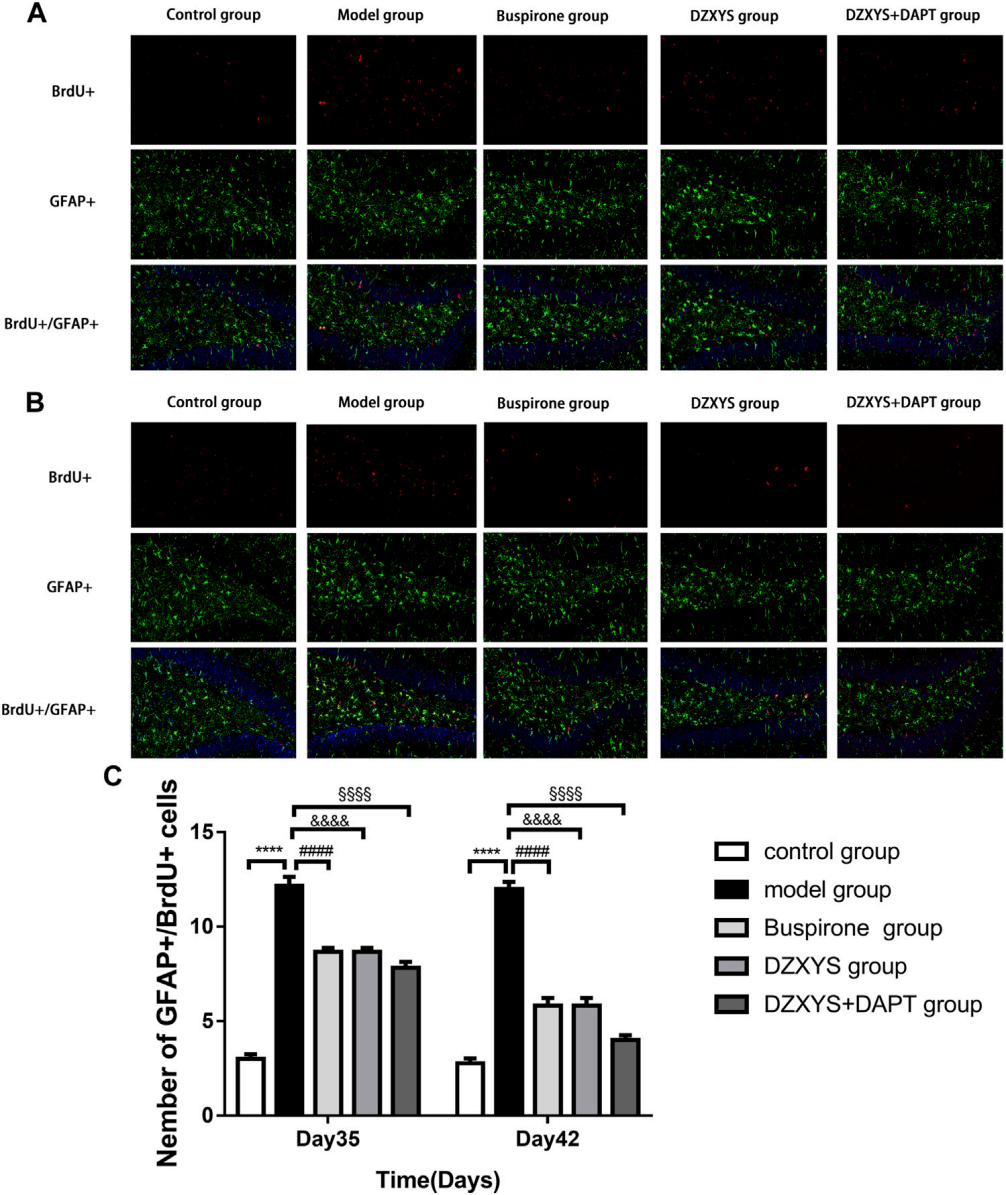
The number of newborn astrocytes in the dentate gyrus of the hippocampus in each group. BrdU (red) labeled newly formed cells, GFAP (green) labeled astrocytes **(A)** The condition of newborn astrocytes on the 35th day **(B)** The condition of newborn astrocytes on the 42nd day **(C)** The number of new GFAP positive cells in the dentate gyrus on the 35th day and 42nd day. ****p < 0.0001, model group vs. control group; ####*p* < 0.0001, Buspirone group vs Model group; &&&&*p* < 0.0001, DZXYS group vs. model group; §§§§*p* < 0.0001, DZXYS + DAPT group vs model group.

To observe the differentiation of newborn NSCs into neurons (Zupanc et al., 2019), we double-labeled with anti-BrdU (red) and NeuN (neuron specific protein; green) (Gusel’nikova and Korzhevskiy, 2015) antibodies. After 7 days (Day 35) and 14 days (Day 42) of drug intervention, the number of newborn neurons in the DG of the hippocampus in each group was significantly different between the groups (Figures 8A–C). The Bonferroni post hoc test showed that the model group was significantly lower than the control group (*p* < 0.0001), and the number of newborn neurons in buspirone, DZXYS, and DZXYS + DAPT was significantly higher than that in the model group (*p* < 0.0001, *p* < 0.0001, *p* < 0.0001; *p* < 0.0001, *p* < 0.0001, *p* < 0.0001, respectively). Two-way ANOVA showed that the number of newborn neurons in the DG area had a significant processing effect (F_4,20_ = 50.39, *p* < 0.0001) and a significant time effect (F_1,20_ = 11.64, *p =* 0.0028). To observe the differentiation of neonatal NSCs into astrocytes in the rat hippocampus, we double-labeled them with antibodies against BrdU (red) and GFAP (a specific marker of astrocytes; green) (Baba et al., 1997). There was a significant difference in the number of new astrocytes in the DG area of the hippocampus between groups on the 35th and 42nd days after modeling (Figures 9A–C). Bonferroni post hoc analysis showed that the number of new astrocytes in the model group was significantly higher than that in the control group (*p* < 0.0001), and it was significantly lower than that in the model group after buspirone, DZXYS, and DZXYS + DAPT intervention for 7 days (Day 35) and 14 days (Day 42) (*p* < 0.0001, *p* < 0.0001, *p* < 0.001; *p* < 0.0001, *p* < 0.0001, *p* < 0.0001). Two-way ANOVA showed that the number of newborn neurons in the hippocampus had a significant processing effect (F_4,20_ = 119.2, *p* < 0.0001) and a significant time effect (F_1,20_ = 39.06, *p* < 0.0001).

### 3.5 DZXYS Downregulated the Expression of Notch1, Hes1, and Hes5 in the Hippocampus

**FIGURE 10:**
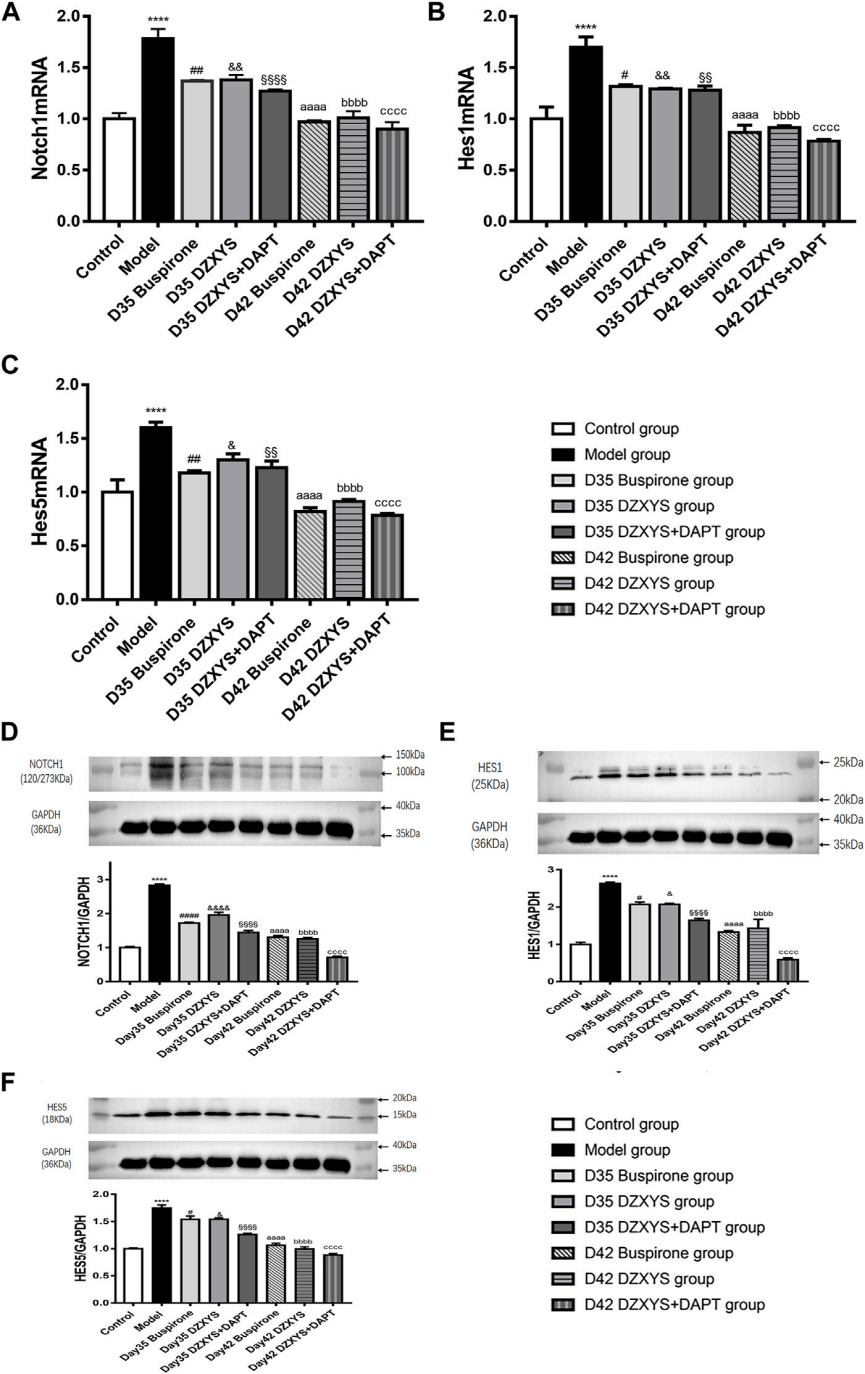
The expression levels of Notch1, Hes1 and Hes5 mRNA transcripts in the hippocampus of rats in each group on the 35th and 42nd days after modeling were measured **(A)** Notch1 mRNA expression **(B)** Hes1 mRNA expression **(C)** Hes5 mRNA expression. Figures D–F show the electrophoretic bands of target protein and GAPDH in hippocampus of rats in different treatment groups, and the relative optical density of target protein in hippocampus of rats in different treatment groups were compared. The results were expressed as the percentage of the optical density of the target protein in the hippocampus of rats, including Notch1 (Figure D)、Hes1 (Figure E)、Hes5 (Figure F). All data are expressed as the mean ± SEM from independent experiments performed in triplicate.****p < 0.0001, model group vs. control group; #*p* < 0.05, ##*p* < 0.01, ####*p* < 0.0001, Day 35 Buspirone group vs Model group; and *p* < 0.05, &&*p* < 0.01, &&&& *p* < 0.0001, Day 35 DZXYS group vs. model group; §§*p* < 0.01,§§§§*p* < 0.0001, Day 35 DZXYS + DAPT group vs model group; aaaa*p* < 0.0001, Day 42 Buspirone group vs Model group; bbbb*p* < 0.0001, Day 42 DZXYS group vs. model group; cccc*p* < 0.0001, Day 42 DZXYS + DAPT group vs model group.

DZXYS upregulated the expression of Notch1, Hes1, and Hes5 in the hippocampus, and there was a significant difference in the expression of Notch1 mRNA in the hippocampus of rats in each group (F_7,16_ = 29.57, *p* < 0.0001) (Figure 10A). The Bonferroni post hoc test showed that the expression of Notch1 mRNA in the model group was significantly higher than that in the control group (1.783 ± 0.093 vs. 1.000 ± 0.058, *p* < 0.0001), and decreased compared to the model group after 7 days of intervention with buspirone, DZXYS, and DZXYS + DAPT (1.370 ± 0.012 vs. 1.783 ± 0.093, *p* = 0.0019; 1.380 ± 0.047 vs. 1.783 ± 0.093, *p* = 0.0025; 1.270 ± 0.017 vs. 1.783 ± 0.093, *p* = 0.0002), and decreased significantly compared with that in the model group after 14 days of drug intervention (0.970 ± 0.017 vs. 1.783 ± 0.093, *p* < 0.0001; 1.010 ± 0.067 vs. 1.783 ± 0.093, *p* < 0.0001; 0.900 ± 0.069 vs. 1.783 ± 0.093, *p* < 0.0001).

There was a significant difference in the expression of Hes1 mRNA in the hippocampus of rats in different treatment groups (F_7,16_ = 24.13, *p* < 0.0001) (Figure 10B). Bonferroni post test showed that the expression of Hes1 mRNA in the model group was significantly higher than that in the control group (1.7 ± 0.10 vs. 1.000 ± 0.115, *p* < 0.0001), and decreased after 7 days of intervention with Buspirone, DZXYS, and DZXYS + DAPT (1.317 ± 0.017 vs. 1.7 ± 0.10, *p* = 0.0144; 1.293 ± 0.007 vs. 1.7 ± 0.10, *p* = 0.0083; 1.28 ± 0.042 vs. 1.7 ± 0.10, *p* = 0.0061). After 14 days of drug intervention (Day 42), it was significantly lower than that in the model group (0.867 ± 0.073 vs. 1.7 ± 0.10, *p* < 0.0001; 0.913 ± 0.019 vs. 1.7 ± 0.10, *p* < 0.0001; 0.783 ± 0.017 vs. 1.7 ± 0.10, *p* < 0.0001).

There was a significant difference in the expression of Hes5 mRNA in the hippocampus of each group (F_7,16_ = 23.86, *p* < 0.0001) (Figure 10C). The Bonferroni post-test showed that the expression of Hes1 mRNA in the model group was significantly higher than that in the control group (1.600 ± 0.052 vs. 1.000 ± 0.115, *p* < 0.0001), and decreased after 7 days of intervention with Buspirone, DZXYS, and DZXYS + DAPT (1.18 ± 0.02 vs 1.600 ± 0.052, *p* = 0.0023; 1.3 ± 0.058 vs. 1.600 ± 0.052, *p* = 0.0494; 1.227 ± 0.064 vs. 1.600 ± 0.052, *p* = 0.0073). After 14 days of drug intervention (Day 42), the expression was significantly lower than that in the model group (0.820 ± 0.035 vs. 1.600 ± 0.052, *p* < 0.0001; 0.913 ± 0.019 vs. 1.600 ± 0.052, *p* < 0.0001; 0.783 ± 0.017 vs. 1.600 ± 0.052, *p* < 0.0001).

There was a significant difference in the amount of Notch1 protein in the hippocampus of rats in different treatment groups (F_7,16_ = 207.3, *p* < 0.0001) (Figure 10D). Bonferroni post hoc analysis showed that the amount of Notch1 protein in the model group was significantly higher than that in the control group (2.828 ± 0.037 vs. 1.000 ± 0.028, *p* < 0.0001, *p* < 0.0001). The amount of Notch1 protein in the model group was significantly lower than that in the model group on the 35th day of the experiment after the intervention with buspirone, DZXYS, and DZXYS + DAPT (1.718 ± 0.035 vs. 2.828 ± 0.037, *p* < 0.0001; 1.957 ± 0.078 vs. 2.828 ± 0.037, *p* < 0.0001; 1.440 ± 0.059 vs. 2.828 ± 0.037, *p* < 0.0001). It continued to decline on the 42nd day of the intervention (1.302 ± 0.049 vs. 2.828 ± 0.037, *p* < 0.0001; 1.255 ± 0.033 vs. 2.828 ± 0.037, *p* < 0.0001; 0.714 ± 0.026 vs. 2.828 ± 0.037, *p* < 0.0001).

There was a significant difference in the amount of Hes1 protein in the hippocampus of rats in different treatment groups (F_7,16_ = 46.57, *p* < 0.0001) (Figure 10E). Bonferroni’s post hoc test showed that the amount of Hes1 protein in the model group was significantly higher than that in the control group (2.633 ± 0.033 vs. 0.995 ± 0.058, *p* < 0.0001). After 7 days of buspirone, DZXYS, and DZXYS + DAPT intervention (Day 35), the amount of Hes1 protein in the model group was significantly lower than that in the model group, especially in the DZXYS + DAPT group (2.067 ± 0.067 vs. 2.633 ± 0.033, *p* = 0.02; 2.067 ± 0.033 vs. 2.633 ± 0.033, *p* = 0.02; 1.643 ± 0.054 vs. 2.633 ± 0.033, *p* < 0.0001). Fourteen days after drug intervention (Day 42), the amount of Hes1 protein in the model group was significantly lower than that in the model group (1.327 ± 0.037 vs. 2.633 ± 0.033, *p* < 0.0001; 1.427 ± 0.239 vs. 2.633 ± 0.033, *p* < 0.0001; 0.587 ± 0.047 vs. 2.633 ± 0.033, *p* < 0.0001).

There was a significant difference in the amount of Hes5 protein in the hippocampus of rats in each group (F_7,16_ = 74.51, *p* < 0.0001) (Figure 10F). Bonferroni post hoc analysis showed that the protein content of Hes1 in the model group was significantly higher than that in the control group (1.751 ± 0.055 vs. 1.000 ± 0.011, *p* < 0.0001). After 7 days of intervention with buspirone, DZXYS, and DZXYS + DAPT (Day 35), the protein content was lower than that in the model group, and the most significant was the DZXYS + DAPT group (1.542 ± 0.059 vs. 1.751 ± 0.055, *p* = 0.0295; 1.542 ± 0.025 vs. 1.751 ± 0.055, *p* = 0.0304; 1.260 ± 0.020 vs. 1.751 ± 0.055, *p* < 0.0001). Fourteen days after drug intervention (Day 42), the protein content of Hes1 in the model group was significantly lower than that in the model group (1.063 ± 0.037 vs. 1.751 ± 0.055, *p* < 0.0001; 0.995 ± 0.039 vs. 1.751 ± 0.055, *p* < 0.0001; 0.882 ± 0.025 vs. 1.751 ± 0.055, *p* < 0.0001).

## 4 Discussion

The purpose of this study was to explore whether the pathogenesis of GAD is related to the abnormal expression of the Notch signaling pathway, and whether the anti-anxiety effect of Danzhi Xiaoyao Powder can promote nerve regeneration in the hippocampus by regulating the Notch signaling. In this animal experiment, we found that after the intervention of DZXYS and DZXYS + DAPT, the food intake and body weight of rats increased significantly compared with the model group; the anxiety-like behavior of the buspirone, DZXYS, and DZXYS + DAPT groups was significantly reversed after 14 days of drug intervention, especially DZXYS + DAPT; immunofluorescence double staining of the hippocampal DG area of rats in each group showed that there was more proliferation of neonatal NSCs in the DG area of rats in the model group, and a large number of them transformed into astrocytes, resulting in obstacles to neurons. After 14 days of treatment with the three groups of drugs, the number of neonatal NSCs in the DG area of rats gradually decreased, the number of neonatal neurons increased significantly, the number of neonatal astrocytes decreased, and the protein expression of key targets of the Notch signaling pathway (Notch1, Hes1, and Hes5) in the hippocampus was gradually lower than that in the model group, and the protein expression of DZXYS + DAPT group was the most significant.

At present, the etiology of GAD is unclear, but it is certain that environmental pressure is an important reason for its onset, repeated fluctuation, and deterioration (Wittchen et al., 2000). Based on this, this study used the uncertain empty bottle drinking water stimulation method (Lin Wenjuan, 2003; Zhang et al., 2020) and chronic restraint stress (Ma et al., 2019) preparation of the GAD rat model. After 21 days of chronic stress stimulation, we found that GAD rats showed significant changes compared with the control group, such as decreased body weight growth rate (Morris, 2019), reduced exploration behavior in the central area of the open field experiment (Deacon, 2013), and significantly reduced exploration time to the open arm in the elevated cross maze (Gasnas, 2021), and there were statistical differences, which proved that the physiological metabolism of the model group was disordered. Moreover, there was anxiety of decreased desire for exploration and fear and timidity, which was consistent with the behavioral performance of anxiety disorder rats prepared by Wenjuan Lin (Lin Wenjuan, 2003) and Shuichi Chiba (Chiba et al., 2012). In addition, during the modeling period, the rats in the model group showed characteristics of depressed expression, easy frightening, dark yellow hair color, loss of luster, curling up in corners, and loose stool (Zhang et al., 2020). It can be seen from the above that the GAD model prepared in our study is accurate and reliable, and the characterization is consistent with clinical symptoms (Luyten et al., 2011).

Observing the changes of food intake of rats after drug intervention, we found that the food intake of rats in the DZXYS and DZXYS + DAPT groups increased significantly compared with the model group after 14 days of drug intervention; It was found that after 7 and 14 days of drug intervention, the body weight of rats in the buspirone, DZXYS and DZXYS + DAPT groups increased steadily compared with the model group, and had a time effect. DZXYS, the combination of DZXYS and DAPT had the most significant effect, indicating that the traditional Chinese medicine DZXYS, and the combination of DZXYS and DAPT can significantly improve appetite, restore normal physiological metabolism, and increase the body weight of rats, which is consistent with the animal experimental results of Yan et al. Yan et al. found that after the intervention of traditional Chinese medicine, the appetite and digestive ability of anxiety and depression rats can be significantly improved. It is speculated that this change is related to the change in the microbial population in the gastrointestinal tract, and its change is closely related to changes in brain metabolites (Qu et al., 2019). This phenomenon reflects the advantages of the multi-target treatment of traditional Chinese medicine.

Through the classical open field experiment, the exploratory behavior and tension of rats in novel and unfamiliar environments were observed, and it was found that the overall activity of rats after modeling showed no significant change compared with the control group, It shows that the limb activity of rats is normal and can move freely (Ann-Katrin et al., 2019), but the activity distance, residence time, and standing times of the central area in the open field were significantly lower than those in the control group after 28 days of modeling. After 7 days (Day 35) and 14 days (Day 42) of drug intervention, the activity distance of the central area in the buspirone, DZXYS, and DZXYS + DAPT groups were higher than those in the model group; the residence time and standing times increased significantly, and the activity of the central area increased most significantly in the DZXYS + DAPT group. This is currently the gold standard for investigating the anxiety of animals at present (Biedermann et al., 2017). The values of OT% and OE% in the model group were significantly lower than those in the control group. After 14 days of drug intervention, the values of OT% and OE% in the buspirone, DZXYS, and DZXYS + DAPT groups were significantly and continuously increased, and the treatment effect and time effect were significant (Figure 5) (Taylor et al., 1985). The above behavioral experimental results show that DZXYS can increase the ability of exploration, reduce the tension, and improve the anxiety behavior of rats. This is consistent with the experimental results obtained by (Zhao et al., 2017). Among them, the anti-anxiety effect of DZXYS + DAPT was better than that of DZXYS alone.

5-HT1A receptor agonists, such as buspirone and tandospirone, have been shown to promote the formation of neurons in the hippocampus of anxiety and depression rats and improve anxiety and depression. As shown in Figure 6, HE staining of rat hippocampus showed that the morphology of neurons in DG area of DZXYS group was slightly improved compared with that of the model group, most cells had clear outline and were closely arranged, only a small amount of nuclear pyknosis and deep staining of cytoplasm were seen, and the number and number of cell layers in DZXYS group were slightly lower than those in the control group (Kai et al., 2020). To understand the anti-anxiety role of DZXYS, we used double immunofluorescence staining (Donaldson, 2015) to observe neurogenesis in the DG area of the hippocampus of rats in each group at 7 days (Day 35) and 14 days (Day 42) after drug intervention. Proliferation of new neural stem cells (NSCs): Compared with the control group, the number of new NSCs in the GAD model group increased significantly. On the 35th day, the number of new NSCs in the DZXYS + DAPT group was lower than that in the model group. On the 42nd day, the number of new NSCs in the hippocampus of the buspirone, DZXYS, and DZXYS + DAPT groups decreased, especially in the DZXYS + DAPT group. This shows that compared with normal rats, neural stem cells in the DG area of the hippocampus of GAD rats had compensatory proliferation, while the proliferation of neural stem cells decreased or was successfully transformed into other neural cells after drug intervention. Proliferation of newborn neurons: Compared with the control group, the number of newborn neurons in the model group decreased significantly. After 7 and 14 days of drug intervention, the number of newborn neurons in the buspirone, DZXYS, and DZXYS + DAPT groups continued to increase significantly, with significant treatment effect and time effect; proliferation of new astrocytes: Compared with the control group, the number of new astrocytes in the DG area of the model group increased significantly. After 7 and 14 days of drug intervention, the number of new astrocytes in the hippocampus of the buspirone, DZXYS, and DZXYS + DAPT groups decreased significantly compared with the model group, and the time effect was significant, especially in the buspirone group which decreased most significantly. According to the above three fluorescent double-standard results, it can be seen that at all time points after the model was built by the model group, a large number of NSCs proliferated and mainly transformed into star-type glial cells, and a small number of them were transformed into new neurons. After DZXYS intervention for 7 days, the proliferation of NSCs decreased, and the number of newly proliferated NSCs in the early stage was transformed into new neurons, The transformation to the new astrocytes decreased significantly and the trend was more obvious with time. We speculate that the neural stem cells in the hippocampus of the model group that received adverse stimulation were over-proliferated and differentiated into astrocytes in large quantities, which could not be transformed into new neurons. Astrocytes are the most widely used glial cells in the brain. An appropriate number of astrocytes can maintain potassium homeostasis around neurons, regulate synaptic transmission, promote the supply of neuronal sugar, and have the ability to divide (Vasile et al., 2017). However, excessive proliferation of reactive astrocytes can form glial scars and secrete growth factors and interleukins, interferons, and other factors that inhibit nerve regeneration, inhibiting the regeneration of neurons and nerve axons (Rossi et al., 2007). Therefore, astrocyte activation plays a “double-edged” effect in the process of central nervous system injury and repair, and it is very important to ensure a steady state of astrocyte number (Lee et al., 2014; Pekny et al., 2016). Neurogenesis in the hippocampus is particularly important in cognition, emotion, and reproductive behavior, and dysfunctional neurogenesis leads to emotional and mental disorders, leading to the emergence of an anxious state. This is similar to Gisele’s observation of neurons in the hippocampus of GAD rats (Dias et al., 2014). DZXYS, especially the combination of DZXYS and DAPT, dynamically reversed the excessive differentiation of neural stem cells into astrocytes, turned into newborn neurons, and improved anxiety behavior, which is similar to the mechanism of hippocampal neuron regeneration in the treatment of depression (Tanti and Belzung, 2013; Hill et al., 2015; Anacker et al., 2018). Therefore, we propose an important question as to how DZXYS dynamically reverses the differentiation direction of newborn neural stem cells at the molecular level.

It is generally recognized that Notch signaling plays an important role in maintaining the proliferation state of neural stem cells, regulating the timing of differentiation, and determining the fate of neural precursor cells (Lutolf et al., 2002; Niessen and Karsan, 2007). Previous studies have shown that the Notch signaling pathway is expressed in the SGZ and SVZ regions of the adult brain (Stump et al., 2002) and participates in adult neurogenesis. The Notch signaling pathway is composed of the Notch receptor, ligand, and CSL (CBF1-suppressor of hairless-lag-1) DNA-binding protein (Ehebauer et al., 2006). This pathway is initiated by the combination of the Notch receptor and its ligand, after which the Notch receptor releases the active Notch intracellular domain (NICD) into the cytoplasm, and the released NICD is transferred to the nucleus to directly regulate the functions of transcription factors CSL [CBF-1, Su(H), and LAG-1] and a series of downstream target genes, including Hes1 Hes5 and Hes related protein (HERP/HEY) genes (Louvi and Artavanis-Tsakonas, 2006). In this study, we observed fluctuations in Notch1, Hes1 and Hes5 mRNA transcription levels and protein expression in the rat hippocampus by PCR and western blotting. The transcription levels of Notch1, Hes1 and Hes5 mRNA in the model group were significantly higher than those in the control group. After 7 and 14 days of drug intervention, Notch1, Hes1 and the expression of Hes5 mRNA decreased gradually, with an obvious time effect. Among them, the transcriptional levels of Notch1, Hes1, and Hes5 mRNA in the hippocampus of rats in the DZXYS + DAPT group decreased most significantly. The changes in the expression of the three proteins detected by western blotting were consistent with the PCR results.

**FIGURE 11:**
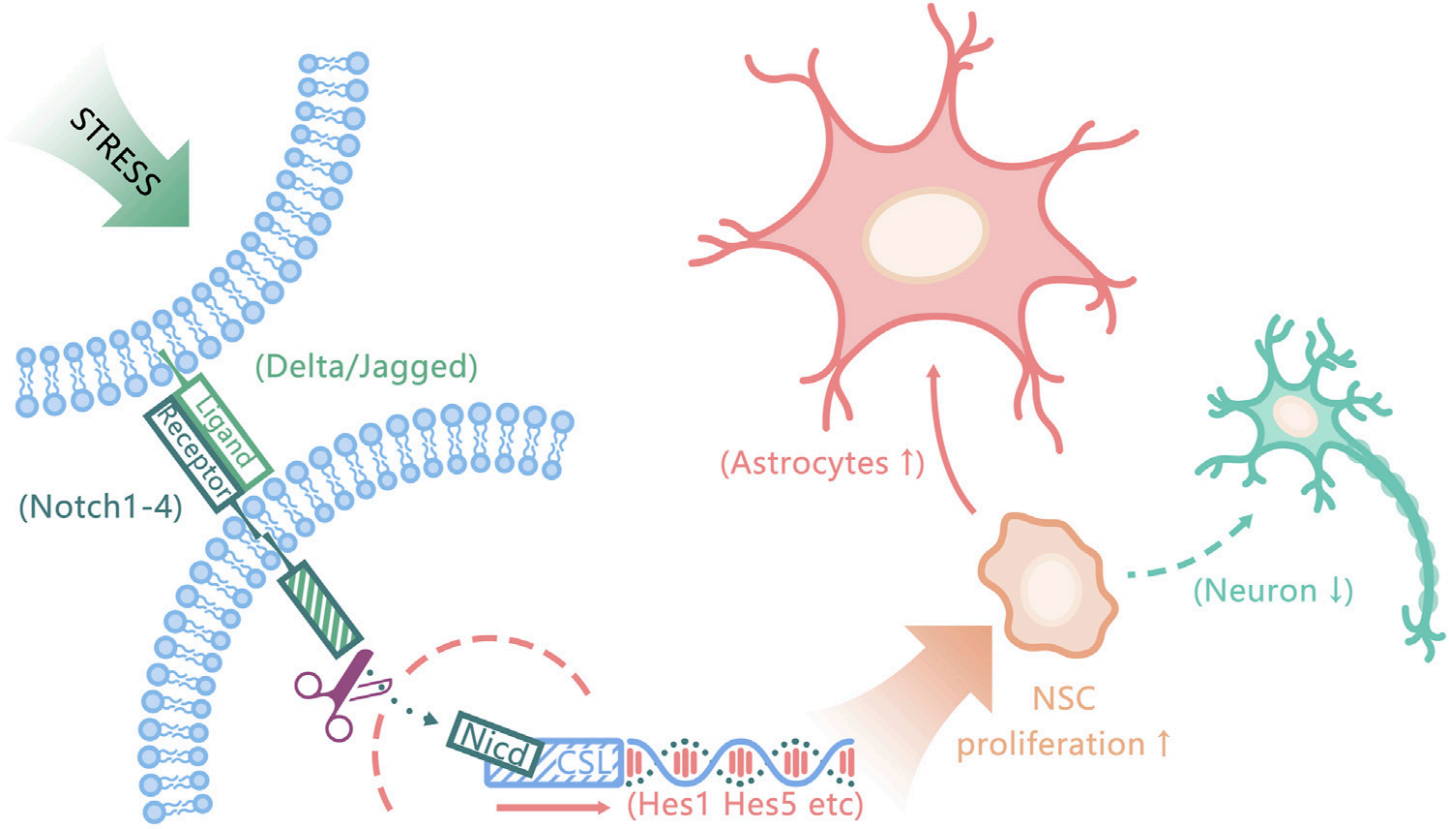
Schematic diagram of molecular pathological mechanism of generalized anxiety disorder. Chronic stress activates Notch signaling pathway, which promotes the proliferation of neural stem cells and massive transformation to astrocytes, resulting in impaired neuronal regeneration (Louvi and Artavanis-Tsakonas, 2006).

Previous studies have shown that the activated Notch signaling pathway can maintain the cellular characteristics of NSCs and inhibit their differentiation into neurons, while promoting their differentiation into astrocytes (Guo et al., 2009; Snyder et al., 2012). The overexpression of Notch1 protein observed in this experiment will lead to a large number of transformations of newborn neural stem cells into astrocytes and neurogenesis disorder (Louvi and Artavanis-Tsakonas, 2006) as shown in Figure 11. Hes1 and Hes5, the main downstream targets of Notch signaling, are essential for regulating neurogenesis. They inhibit the transcription of precursor genes, leading to the inhibition of neuronal differentiation (Ohtsuka et al., 1999), the emergence of new astrocytes also depends on the activities of Notch downstream effectors Hes1 and Hes5 (Givogri et al., 2006). The transcriptional levels of Notch 1, Hes1, and Hes5 mRNA in the model group were significantly higher than those in the control group, which revealed that the deep-seated molecular mechanism of GAD was related to the activation of the Notch signaling pathway and the overexpression of key targets. DZXYS can downregulate the Notch signaling pathway, inhibit the continuous proliferation of neural stem cells, and promote their differentiation into neurons, thus improving the anxiety behavior of rats. DAPT in the DZXYS + DAPT group (*γ*-secretase inhibitors) can inhibit the transmission of Notch 1 signaling, inhibit the activation of the Notch signaling pathway, reduce the excessive proliferation of neural stem cells, and promote their transformation to newborn neurons (Breunig et al., 2007). Therefore, the decrease in protein expression of the main target of the Notch signaling pathway in the hippocampus of rats in the combination group of DZXYS and DAPT was more significant than that in other drug intervention groups. The Notch signaling pathway was most effectively inhibited, the proliferation level of neural stem cells decreased, and then differentiated into a large number of newborn neurons. The anxiety behavior was most significantly improved, which also confirmed that inhibiting the overexpression of the Notch signaling pathway and promoting the regeneration of newborn neurons is the key to alleviating anxiety behavior. Many common active ingredients in DZXYS can regulate Notch signaling pathway and promote neuronal regeneration. It has been found that sodium ferulate combined with bone marrow stromal cells can down regulate Notch signaling pathway and promote neural regeneration after focal cerebral ischemia in rats (Zhao et al., 2013); It was also found that baicalin could down regulate the expression of basic helix-loop-helix (bHLH) protein downstream of Notch signaling pathway and induce the differentiation of human iPS cells into neurons (Morita et al., 2015). At present, mental diseases are less associated with the Notch signaling pathway, and there is a great controversy about how the Notch signaling pathway plays a therapeutic role in limited studies. For example, Guo et al. observed the Notch signaling pathway in the hippocampus of post-stroke depression rats and found that the Notch signaling pathway in the hippocampus of depression rats is inhibited, neuronal apoptosis and necrosis are increased, and there are few newborn neurons; after intervention with antidepressants, the Notch signaling pathway was activated, and the protein expression of key targets (especially Hes1 and Hes5) increased, which promoted the increase of newborn neurons and alleviated depressive behavior (Guo et al., 2009). We speculate that the differential expression of the Notch signaling pathway protein is related to the special pathological state of post-stroke brain tissue in this study.

The neuronal hypothesis has been the focus of international psychiatry research in the past 10 years, and it is also one of the most challenging research fields. For the treatment of depression, experimental and theoretical research on promoting hippocampal neuron regeneration and improving depressive symptoms has gradually matured. Whether it can effectively promote hippocampal neuron generation has become an important new target to determine the effectiveness of antidepressants. As a chronic mental disease (Fenton, 1996), generalized anxiety disorder (GAD) usually precedes depression. Some experts believe that the emergence of depression may indicate that compensatory activities cannot protect themselves from the chronic pressure imposed by GAD and eventually develop into depressive disorder (Kessler et al., 1999; Maron and Nutt, 2017). If GAD symptoms can be reversed in the early stage of the disease, it can not only greatly alleviate the great mental pain experienced by patients with GAD, but also avoid the occurrence of depression to a certain extent. DZXYS has been widely used in the clinical treatment of GAD and has a definite curative effect on the disease, but it has not received extensive international attention because of its unclear treatment mechanism (Xu et al., 2020). Through this study, we found that the deep-seated molecular mechanism of DZXYS promotes neuronal regeneration in the hippocampus to treat GAD by downregulating the Notch signaling pathway, which not only opens up new ideas for the development of more effective anti-anxiety drugs in the future, but also lays a foundation for further exploration of the effective components in the prescription. In addition, imaging tools can be developed to detect the level of adult hippocampal neurogenesis in patients with GAD, so that the degree of increased adult hippocampal neurogenesis can become a new biomarker for the efficacy of anti-anxiety drugs.

### 4.1 Summary and Limitations of Current Study

This study shows that the excessive up regulation of Notch signaling pathway in the hippocampus of GAD rats leads to a large number of proliferation and differentiation of neural stem cells into astrocytes, and neuronal regeneration is impaired; DZXYS can play a positive role in neurogenesis by inhibiting the overexpression of Notch signal pathway in hippocampus, promoting neuronal regeneration in hippocampus and inhibiting the differentiation into astrocytes, and has a significant time effect.

There are still three deficiencies in the design of this subject. Firstly, the experimental model was prepared using the chronic mild stress paradigm. Therefore, at the end of 21 days, we should not only use OFT and EPM to detect the formation of anxiety behavior in rats, but also use sucrose preference test to eliminate the depression phenotype (Hyeonwi et al., 2019), so as to eliminate the interference of depression phenotype rats to the experiment, In the future experimental design, we will supplement the sucrose preference experiment; Secondly, we only mechanically observed that DZXYS can downregulate the Notch signaling pathway, promote the increase of neuron number, but ignored the important part of neuronal synaptic plasticity, which is an important part of nerve regeneration. In the next experimental study, we need to focus on whether DZXYS can regulate NMDA receptor to a certain extent after intervening in Notch signal pathway in anxiety rats, promote neuronal synaptic plasticity without inducing its excitotoxic effects (Corlew et al., 2008; Moreau and Kullmann, 2013; Deutschenbaur et al., 2016). At the same time, in the next experiment, we will co locate Notch1 with BrdU, Nestin and NeuN by immunofluorescence in various regions of hippocampus, observe the specific expression changes of Notch1, and find the specific regions with elevated Notch1 level. At last, the abnormal expression of a single Notch signaling pathway is not sufficient to reveal the pathogenesis of generalized anxiety disorder. The therapeutic effect of traditional Chinese medicine is also multi-target and multi-channel. We should seek other effective multi-signal transduction pathways and explore the cross connection and interaction between various pathways to further explore the pathogenesis of GAD and explore the deep treatment mechanism of traditional Chinese medicine.

## Data Availability

The original contributions presented in the study are included in the article/Supplementary Material, further inquiries can be directed to the corresponding authors.

## Abbreviations

BrdU, 5-bromo-2-deoxyuridine; CE, closed arm entry times; CMC-Na, sodium carboxymethyl cellulose; CT, time in the closed arm; DZXYS, Danzhi Xiaoyao Powder; DAPT, *γ*-secretase inhibitor; DG, dentate gyrus; EPM, elevated plus maze; GAD, generalized anxiety disorder; NSCs, neural stem cells; OE, open-arm entry times; OFT, open-field test; OT time in the open arm.
